# Exploring the Chemical Properties and Biological Activity of Four New Zealand Monofloral Honeys to Support the Māori Vision and Aspirations

**DOI:** 10.3390/molecules27103282

**Published:** 2022-05-20

**Authors:** Claire Zucchetta, Wally Tangohau, Aaron McCallion, Derrylea J. Hardy, Andrea Clavijo McCormick

**Affiliations:** 1 School of Agriculture and Environment, Massey University, Tennent Drive, Palmerston North 4474, New Zealand; claire.zucchetta@gmail.com; 2 Te Pumautanga o Te Arawa Trust, 1196 Haupapa Street, Rotorua 3010, New Zealand; wallyt@tpota.org.nz (W.T.); aaron@wakadigital.co.nz (A.M.); 3 School of People, Environment and Planning, Massey University, Tennent Drive, Palmerston North 4474, New Zealand; d.j.hardy@massey.ac.nz

**Keywords:** native honeys, Aotearoa New Zealand, indigenous development, sustainable land use, bee products, ethnobotany, mātauranga Māori, indigenous knowledge

## Abstract

Honey production and export are significant contributors to the Aotearoa New Zealand economy, generating over 400 million dollars in revenue. Its main export is mānuka (*Leptospermum scoparium*) honey, which has a high commercial value due to its medicinal properties that are linked to its unique chemical composition. The compound methylglyoxal (MGO) has been identified as the main floral marker and is used as a quality indicator, often labelled as unique mānuka factor (UMF). However, the high demand for mānuka honey creates pressure on beekeepers and may have negative ecological consequences by favouring extensive mānuka monocultures to the detriment of other native species. There are other honeys native to New Zealand, such as kāmahi (*Weinmannia racemosa*), kānuka (*Kunzea ericoides*), rātā (*Metrosideros robusta*) and rewarewa (*Knightia excelsa*), that also have medicinal properties; however, they are less well known in the local and global market. Indigenous Māori communities envision the production and commercialization (locally and internationally) of these honeys as an opportunity to generate income and secure a sustainable future in alignment with their worldview (Te Ao Māori) and values (tikanga Māori). Diversifying the market could lead to a more sustainable income for beekeepers and reduce pressure on Māori and the conservation land, while supporting indigenous communities to realize their vision and aspirations. This manuscript provides an extensive review of the scientific literature, technical literature and traditional knowledge databases describing the plants of interest and their traditional medicinal uses (rongoā) and the chemical properties of each honey, potential floral markers and their biological activity. For each honey type, we also identify knowledge gaps and potential research avenues. This information will assist Māori beekeepers, researchers, consumers and other stakeholders in making informed decisions regarding future research and the production, marketing and consumption of these native monofloral honeys.

## 1. Introduction

The production of honey and related products for domestic consumption and export is an important economic activity in Aotearoa New Zealand (NZ). In 2019/20, total NZ honey exports were $425 m, showing a 28% increase from the previous period (2018/2019), with the main export markets being China, the United States, Japan, Germany and Australia [1]. Among these exports, mānuka (*Leptospermum scoparium*) honey is the most prominent and has been reported to have exceptional antioxidant and antimicrobial properties [2]. However, non-mānuka honey exports, with a market value of $48 m, represent 11% of the export market value [1].

Mānuka is unique to NZ and was recognised in early Māori traditions as a taonga or “treasure” due to its wide variety of uses [3]. The medicinal benefits of mānuka honey reflect its high market value, with an average export unit price of $53.13/kg for monofloral mānuka and $31.33 for multifloral mānuka in 2020/2021 [4]. These medicinal benefits have been linked to its unique chemical composition: mainly, the presence of a compound called methylglyoxal (MGO) [5]. MGO concentration is commercially used together with the presence of other compounds, such as leptosperin and dihydroxyacetone (DHA), to describe the quality and purity of honey as the unique mānuka factor (UMF) [6]. However, a variety of native honeys (other than mānuka) are produced in NZ by indigenous Māori producers and small apiaries—for example, kāmahi (*Weinmannia racemosa*), kānuka (*Kunzea ericoides*), rātā (*Metrosideros robusta*) and rewarewa (*Knightia excelsa*)—all of which have unique organoleptic properties (colour, taste, smell, viscosity, etc.); however, these are often mixed together into “bush honeys” of low commercial value.

In contrast to the high value of mānuka honey, the average price paid to beekeepers for non-mānuka honey dropped by 30–50% in the 2019–2020 season, continuing the decline of the previous three years, from a range of $10–14/kg in 2016–2017 to $2.50–5.50/kg [4]. The discrepancy between mānuka honey and the prices of other honeys has pushed the industry towards a greater production of mānuka honey [7], which involves financial pressure for apiculturists and the environmental impacts of large mānuka crops being planted to sustain the demand. Some Māori honey producers see an opportunity to explore the commercial potential of non-mānuka monofloral honeys and have led this work to better understand the properties and biological activities of these honeys. For this review, academics from Massey University and representatives from the Te Pumautanga o Te Arawa Trust (TPT) collaborated to gather information from the available scientific literature, postgraduate theses, technical reports and traditional Māori knowledge (mātauranga Māori), with the goal of supporting the TPTs vision and aspirations for the Te Arawa iwi, hapū and whanau (tribes/clans/families) as described below:

Our vision is having Tino Rangatiratanga (sovereignty) over our lands, our waterways and our natural resources and how our traditional knowledge belief system provides insights into sustainable development, conservation protection and biodiversity. We believe that all parts of the environment have a life force. Over generations, we have developed systems for gaining a reliable understanding of the world around us. These foundations and understandings have been passed down through the generations through practical actions, awareness and the correlation of activities in relation to the cycle and phases of the moon and the tides. It is about storytelling and mapping. We were the practitioners who are now attempting to validate our traditional knowledge with Western science. This research must be grounded in collectivising the following principles operating in unison: Kawa, guiding philosophy; tikanga, supportive practice; and Kaupapa, collective endeavour. We hold the belief that, through intermingling Western knowledge with mātauranga Māori (traditional knowledge), our people will prosper and fulfil the following aspirations:

*Developing sustainable land use opportunities for native forests*—The Iwi (in this case, the Te Arawa Iwi) aims to explore the considerable potential of building high value niche businesses utilising forests and mātauranga Māori to create value and to ensure that any use of forest resources is sustainable.

*Community and Intergenerational Wellbeing*—The Te Arawa Iwi is keen to establish commercial entities that are sustainable, resilient to climatic changes and contribute to the intergenerational wellbeing of our people. An overarching consideration for the Iwi is to develop enterprises, markets and industries that are informed and align with the Te Ao Māori (Māori world view) and the specific aspirations of the Iwi.

*Unlocking the science and innovation potential of Māori knowledge, resources and people for the benefit of Aotearoa New Zealand*—This work enhances collaboration and linkages between mātauranga Māori (traditional knowledge) and Western science. Future projects related to this research will foster mātauranga Māori associated with the kaitiakitanga (guardianship) of forests and other taonga in the rohe (the ancestral land associated with each Iwi), promote the use of honeys in rongoā (traditional medicine) and support the development of native honey enterprises for Iwi, hapū and whanau (tribes/clans/families).

## 2. Definition of Honey and Monoflorality

As outlined in the Codex Alimentarius Standard 12 [7], honey is “the natural sweet substance produced by honeybees from the nectar of plants or from secretions of living parts of plants or excretions of plant sucking insects on the living parts of plants, which the bees collect, transform by combining with specific substances of their own, deposit, dehydrate, store and leave in the honeycomb to ripen and mature.” Under the Codex, a honey can be considered monofloral if it comes wholly or predominantly from a particular source [7].

The monoflorality of honey determines its market value; therefore, it is of extreme importance for beekeepers to be able to assess the botanical origin of their honey. Beekeepers can identify the floral sources of honeys by their colour, texture, odour and taste. For their own honeys, they know where they place the hives and thus the predominant botanical species that contribute to their honey. However, for commercial purposes, this is often insufficient and other tests, such as pollen analysis (melissopalynology), are usually performed on the samples to determine their botanical origin. The use of pollen analysis has proven to be challenging in the NZ context. In 2013, the Ministry of Primary Industries in NZ identified the limitations of pollen counts to determine mānuka honey monoflorality, since mānuka pollen is indistinguishable from that of kānuka under light or scanning electron microscopes [8]. Molan [9] reviewed the limits of this method and its applicability to NZ honeys. The author noted that NZ native flora did not evolve to rely on honeybee pollination; therefore, bees can access the nectar without touching the pollen (e.g., in rewarewa), suggesting that pollen analyses may not be reliable for NZ native honeys. In recent years, a substantial body of research has been developed to identify floral markers that establish the origin and quality of NZ native honeys, e.g., MGO in mānuka honey (reviewed in [10]). In the following sections, we will explore four native plants of interest for honey production, their traditional uses and what is known about their honey in terms of its chemical markers and biological activity.

## 3. The Native Plants of Interest and Their Traditional Uses in Rongoā (Traditional Māori Medicine)

Below, we will introduce the different native plants of interest with a particular emphasis on their Māori traditional medicinal uses.

**Figure 1:**
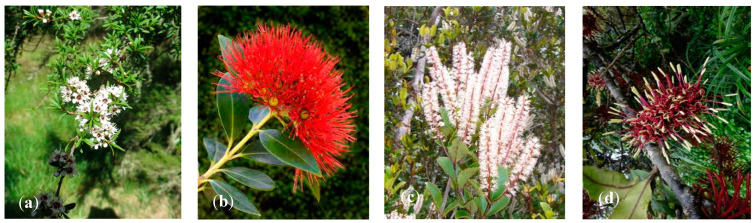
Native NZ plants included in this study: (**a**) Kānuka (*Kunzea ericoides*), photo by Kathy Warburton, source Wikimedia Commons; (**b**) southern Rātā (*Metrosideros umbellata*)*,* photo by Sid Mosdell, source Fickr; (**c**) Kāmahi (*Weimania racemose*), photo by Alan Liefting, source Wikipedia; (**d**) Rewarewa (*Knightia excelsa*), photo by John Barkla, source Wikimedia commons. All under creative commons licence.

Kānuka (Figure 1a) is the common Māori name for ten botanical species found in NZ, with certain species located in a very narrow distribution range (e.g., Great Barrier Island kānuka, *Kunzea sinclarii*; Three Kings Islands kānuka, *Kunzea triregensis*). Kānuka is not found in south Westland or the southern districts of the South Island and does not tolerate wet soils or sub-alpine areas [11].

New Zealand has 11 rātā species (Figure 1b): three tree species, one shrub, and six climbing species [12]. Northern rātā (*Metrosideros robusta*) is one of NZ’s tallest flowering trees. It usually begins life as an epiphyte high in the forest canopy. Its roots grow down to the ground, finally enclosing the host tree and producing a huge tree. It is found throughout NZ’s North Island and in the South Island, down to about Westport in the South Island. Northern rātā is found in coastal and lowland forests, occasionally extending to montane forests in some parts of the country. It has been reported that northern rātā can hybridize with pōhutukawa (*Metrosideros excelsa*) [13].

Two species of kāmahi (Figure 1c) are found in NZ (*Weinmannia sylvicola* Sol ex A. Cunn and *W. racemosa*). The former occurs only on the North Island. Kāmahi is a widespread and common tree found in disturbed habitats in coastal and lowland to montane forests, often becoming locally dominant in higher altitude montane forests in the higher ranges of the North Island and western South Island [14].

Rewarewa (*Knightia excelsa*; Figure 1d) is an endemic monotypic genus. It is a common tree to coastal, lowland and lower montane shrublands found on the North Island; however, it is confined to the Marlborough Sounds in the South Island [15].

All these plants were used in earlier times by Māori healers. Nowadays, they are still used as part of rongoā—traditional Māori medicine—for their various qualities. Manaaki Whenua Landcare Research created a database (Ngā Tipu Whakaoranga, “the plants that sustain us”) [16] containing fully referenced, detailed information on how Māori used plants in Aotearoa New Zealand, particularly before the arrival of Europeans. The enormous body of work is based on information taken from the written record, mostly published (i.e., books, articles and newspapers) and some unpublished (i.e., manuscripts and letters). The following section is based on that database and all the references are available on their website.

Table 1 summarizes the diverse traditional medicinal uses of the five native honeys of interest. Altogether, they are used to ease pain, promote healing and treat a broad range of afflictions. The main plant part used is the bark, which is boiled and placed topically on the affected area, or even mixed with elements from other plants. Leaves and young shoots are also reported to be used in rongoā as infusions or are chewed and applied locally. It is worth noting that mānuka and rātā are the plants with the most reported therapeutic uses involving all the plant’s parts (bark, leaves, fruit, sap, nectar, etc.).

The nectar of several blossoms, including rewarewa (*K. excelsa*) and rātā (*Metrosideros* spp.), used to be collected and eaten by Māori who picked the flowers in late spring and tapped them onto the inside of a gourd vessel [16]. Wai kaihua (nectar) from northern rātā has been reported to be used for sore throats [16]. It is referred to as “honey” even though the reference does not mention honeybees; it is therefore possible that Māori used the raw nectar or processed it in a delicacy similar to the honey produced by honeybees.

Although there is no record of the traditional uses of honey, one could hypothesize that, after the introduction of hives to Māori people, knowledge was shared regarding the properties of honeys. It is yet to be verified whether Māori started using honey similarly to other civilizations throughout history. As they were already using nectar, it is likely that they would use the honey in a similar way; however, more research investigating the traditional use of these honeys is needed to support this hypothesis.

## 4. Characterization of Native Honeys and Their Biological Activity

Honey can be characterised based on its organoleptic properties (taste, colour, smell, etc.) and its physicochemical properties (pH, conductivity, mineral content, etc.). The presence of unique compounds, known as floral markers, may be helpful in elucidating the honey’s botanical origin, and some of these markers can be associated with the biological activity of the honey (e.g., antibacterial, anti-inflammatory or immunostimulant activity).

In 1989, Zumla and Lulat stated in their review on the traditional uses of honey around the world: “the time has now come for conventional medicine to lift the blinds off this ‘traditional remedy’ and give it its due recognition” [17]. Ever since, there has been growing interest on the diverse therapeutical uses of honey. Honey as a medicine is an affordable and harmless alternative, with no reported serious side effects compared to the synthetic chemicals used to treat the same afflictions [18,19]. Several medical-grade honeys are already available on the market (e.g., Medihoney©, Revamil© and Honevo©); the requirements of medical-grade honey are discussed in Hermanns et al. [20].

In the next section, we will review the known physicochemical parameters of the five native honeys of interest, their potential floral markers and tested biological activity.

### 4.1. Kāmahi Honey

#### 4.1.1. Properties and Floral Markers

Kāmahi honey is a light-coloured honey with a pH of 4.78, a conductivity of 0.68 mS/cm and a moisture content of 17 g per 100 g of fresh weight. Its mean total mineral content is 1930 mg/kg, with potassium as the main mineral component (1770 mg/kg) [21]. These measurements are consistent with those from Senanayake [22] and Langford et al. [23], which were completed using the hydroxymethyl furfural (HMF; used as an indicator of heat and storage changes in honey) content and carbohydrate contents (fructose, glucose and maltose). Although the HMF and carbohydrate contents differ slightly between the two studies, this may indicate seasonal/geographical variations in the kāmahi honeys tested. It is worth noting that assessing floral integrity via pollen analysis is difficult for kāmahi honey samples, as they generally present other sources of pollen [24].

A first list of diethyl ether extracts of kāmahi honey (n = 11) is available from Hyink [25], including the associated GC/MS data. Broom et al. [26,27] reported the presence of unique chemical markers in kāmahi honey—nor-sesquiterpenoids named **kamahines A, B** and **C**—and their structure and potential precursors. Broom [28] investigated kāmahi plant parts and nectar in their search for kamahines or precursors; however, they could not find any in the samples they analysed, leaving the chemical origin of kamahines unexplained.

Ede and Wilkins [29] identified another nor-sesquiterpenoid: **meliracemoic acid**. Broom’s thesis [28] gives more details on the methodology that allowed them to determine the stereochemistry and absolute configuration of kamahines. It was later confirmed that kāmahi honey is characterized by the presence of **meliracemoic acid** and **kamahines A–C,** and these compounds are typically present at average quantities of 14 and 73 mg/kg of honey, respectively. In addition, another study reported that **2,6-dimethylocta-3,7-diene-2,6-diol** was present in an average quantity of 31 mg/kg [22]. These results are consistent with those of Hyink [25].

Finally, similar to previous studies, **an unidentified 266 Da compound** was also reported, which suggests an oxygenated degraded carotenoid-like compound analogous to kamahines. This compound, which was one of the most discriminant in a linear discriminant analysis, was only found in kāmahi honey [24]. It could be related to a finding reported by Spiteri et al. [30] of an NMR marker (1.975–1.9 ppm) for kāmahi-labelled honey that had yet to be identified.

Senanayake studied the composition of kāmahi honeys (n = 10) and provided the average concentrations of compounds in methylated diethyl ether extracts of kāmahi honeys ([22], Chapter 3, Table 3.4). However, the presence of contaminants due to the extraction method led some to criticise the reliability of these results. In another study, Goss [24] analysed kāmahi honey with the aim of providing peer-reviewed standards of its chemical composition using previous work, including Senanayake’s. The results showed the presence of **2,6-dimethylocta-3,7-diene-2,6-diol**, which was previously reported in kāmahi honey, and another potential marker (**2,2,6-trimethylcyclohexane-1,4-dione**), which was absent from the other NZ honeys used for comparison.

Another lead explored by Sun [31] was the presence of glycosides—molecules composed of a sugar and another functional group, linked together by a glycosidic bond. The hypothesis stated that due to the high sugar content of honey, it could be expected that some molecules would be present in glycoside forms at one point; however, these forms would be degraded through chemical processes during the honey’s life, storage and use. Using α- and β-glucosidase after an acid treatment, various NZ honeys, including kāmahi honey (n = 1), were analysed and their chromatograms were compared to those of untreated honeys. The treated kāmahi sample displayed a higher level of **4-hydroxy-3-methyl-2-*trans*-pentenedioic acid**, suggesting that this compound may be present as a glycoside. In the same paper, a list of extractives of tōwai honey (*Weinmannia silvicola*) is available, allowing for comparison within the *Weinmannia* family.

In the study by Petchell [32], honey from various floral sources was grouped via a probability plots method after a solid-phase mass extraction (SPME) analysed by GC-MS. Probability plots show the abundance of each compound across the *x* axis (the semi-quantitated concentration), and the percentage of samples falling within a region (based on a normal probability distribution) up the *y* axis. This method allows the identification of the discriminant compounds and their concentrations. In the results, kāmahi honey was characterised by the absence of **pantoyl lactone** and an abundance of **4-methyl-5H-furan-2-one**. This method also allowed the authors to confirm multiflorality when honeys were misclassified (e.g., rewarewa honey grouped as a kāmahi honey due to a high kāmahi contribution, previously detected via pollen analysis).

To identify the floral source, a multistep determination flowchart was proposed by Goss [24] (Chapter 6, § 6.4.1) based on the NIR spectra and other characteristics (e.g., conductivity, colour, glucose content). Another classification flowchart based on an SPME and GC-MS analysis was presented by Petchell [32] (Chapter 3, § 3.5).

Langford et al. [23] used selected ion flow tube-mass spectrometry (SIFT-MS) to characterize monofloral NZ honeys based on their aroma signature, including kāmahi (n = 5). After their analysis, they were able to list 22 compounds and their concentrations (in μg/L) that were present in the samples. Using their data, they performed two multivariate analyses and successfully clustered the honeys by botanical origin. It was stated that “the most effective were the compounds with the highest volatility: methanol, ethanol and acetaldehyde”. The authors noted that these compounds could be considered less reliable in postharvest analysis due to the fermentation that occurs in honey. After removing those volatile compounds, **dimethyl sulphide** yielded the highest discriminatory power and allowed a second analysis, in which the clustering of kāmahi honeys was more scattered but still significant and distinct from other clusters.

In another study, fluorescence analyses were performed to discriminate honey from various floral sources, in which only two samples of kāmahi honey (one from the North Island and one from the South Island) were analysed. Bong et al. [33] used pairs of excitation-emission wavelengths to identify pure mānuka and kānuka honeys as well as blended mānuka/kānuka honeys. However, this method failed to group kāmahi honeys in a clear and distinct cluster, supporting the inadaptability of the fluorescence method for kāmahi honey at the chosen wavelengths.

#### 4.1.2. Biological Activity

##### Antibacterial Activity

Molan et al. [34] compared the antibacterial activities of native honeys, including kāmahi honey (n = 3 pure kāmahi, n = 1 blended kāmahi honey), on *S. aureus* with an agar diffusion assay. The results showed that kāmahi was active against the bacteria; however, it was less active than kānuka and mānuka honeys. It expressed a greater inhibitory zone than several other honeys, including rewarewa, clover and rātā, which displayed very small inhibitory zones. Due to the low number of samples, the authors acknowledged the need to reassess the significance of their results.

In 1991, Allen et al. continued the investigation on the antibacterial activity of NZ honeys using phenol equivalents in an agar diffusion assay [35]. Once again, kāmahi honey expressed some level of antibacterial activity against the same bacteria (*S. aureus*). Sixty-two per cent of kāmahi samples were above the overall average activity of the 345 honeys tested. Kāmahi, however, was not one of the most active honeys and displayed an average antibacterial activity.

In another study, Brady et al. [36] investigated the antibacterial and antifungal activity of non-mānuka NZ native honeys. They tested two bacteria, *Escherichia coli* and *S. aureus*. Out of the 14 samples tested, kāmahi honey was among the most sampled honey types alongside honeydew (n = 18), nodding thistle (n = 34), rewarewa (n = 22) and thyme (n = 18). Against *S. aureus*, nine of the fourteen samples of kāmahi yielded an activity of 13.7% (consistent with Allen et al. [35]), which was lower than the mānuka standard (28.4%). The results for the antibacterial activity against *E. coli* showed a greater minimum inhibitory concentration (% honey *v*/*v*) than the mānuka standard for eight out of fourteen samples—23.4% vs. 6.3%—meaning that kāmahi honey loses its antibacterial effect when diluted above 23.4%. No kāmahi honey showed non-peroxide activity.

The antibacterial activity of kamahines was investigated at the Chemical Department of the University of Canterbury by “Ms G Ellis” (as cited in [28] Chapter 4, § 4.4.8); however, the primary source could not be found. The secondary source reported that kamahines did not display any antibacterial activity against various bacteria species (*E. coli*, *S. aureus*, *P. aeruginosa* and *B. subtilis*), although this cannot be confirmed.

The mechanisms and chemical pathways behind the antibacterial effect of honey are still under investigation and are most likely very dependent on the bacteria and floral source of the honey. For example, Lu et al. [37] explored the effect of honey on the cell morphology of four bacteria species. They reported various and diverse responses, including changes in cell length, cell lysis and DNA appearance. The diversity of effects was suggested to be evidence of a variety of regulatory circuits expressed by the bacteria in response to the stress of honey treatment.

##### Antifungal Activity

The only study that investigated the antifungal activity of kāmahi honey (n = 14) adapted the agar diffusion method to assess the effect of honey on the fungus *Trichophyton mentagrophytes* var. *mentagrophytes* and a yeast species, *Candida albicans* [36]. The first species causes fungal skin infections in humans and animals. According to the results, eight out of fourteen kāmahi samples were active with a mean zone of inhibition of 13.8 mm, lower than mānuka’s (18.4 mm). Not all the tested honeys were antifungal; however, kāmahi was among the active samples. The results also showed that *C. albicans* was not affected by any of the tested honeys and was even favoured by their sugar content.

As stated earlier, “Ms G Ellis” (as cited in [28] Chapter 4, § 4.4.8) investigated the antifungal activity of kamahines. She reported that they did not display any antimicrobial activity against *C. albicans*, *T. mentagrophytes* and *C. resinae*. Since the primary source was unavailable, this information remains to be confirmed.

#### 4.1.3. Summary and Research Gaps

From the literature, it is clear that **meliracemoic acid, kamahines A–C** and **2,6-dimethylocta-3,7-diene-2,6-diol** are confirmed floral markers for kāmahi honey. Senanayake [22] and Goss [24] contributed to the determination of kāmahi honey’s composition; however, the presence of contaminants resulting from the extraction method used leads to questions about their results. They do, however, provide a good starting point in the chemical characterisation of kāmahi honey that can be built upon in further investigations. For example, it would be interesting to see if the **unidentified 266Da** [24] compound is the same as that causing the NRM marker (1.975–1.9 ppm) reported by Spiteri et al. [30]. **2,2,6-trimethylcyclohexane-1,4-dione** seems to be absent from other native honeys and could be studied as another floral marker within NZ.

Fluorescence analyses were unable to distinguish kāmahi from other honeys; however, chemical multivariate discriminations succeeded in clustering kāmahi honeys together based on the absence/presence of certain chemical compounds, the concentration of said compounds and also several characteristics, including colour, conductivity and carbohydrate contents.

The antibacterial and antifungal activity of kāmahi honey has been largely understudied; its effects have only been investigated in two bacteria species, one yeast and one fungus. The available published work shows potential antibacterial activity against *S. aureus* and *E. coli*, and antifungal activity against *T. mentagrophytes* var. *mentagrophytes*. Further exploration of its biological activity against other microorganisms is needed. It would also be valuable to investigate antiviral, anti-inflammatory, antioxidant, antitumor and immune-regulatory activities to assess the potential of a medical-grade kāmahi honey.

### 4.2. Kānuka Honey

#### 4.2.1. Properties and Floral Markers

Due to its resemblance to mānuka honey, a great deal of the available studies focus on how to differentiate kānuka from mānuka. A review by Schmidt et al. [38] sums up the main discoveries and ways to distinguish mānuka from kānuka and also highlights the specificities of kānuka. The paragraphs below are based on the same literature but are presented differently to complement, without redundancy, the work of Schmidt et al. [38].

A list of the extractives of kānuka monofloral honey (n = 2) is available in papers by Tan et al. [39,40]. The authors analysed ether extracts made from aqueous solutions of mānuka, kānuka and clover honeys with a continuous liquid/liquid extractor. After methylation, the extractives were identified by 1H and 13C NMR analysis. They identified 56 of the 61 detected compounds and found that kānuka presented a high concentration of aromatic acids.

An analysis of the phenolic compounds and methylglyoxal (MGO) in mānuka and kānuka (n = 4) NZ honeys, including one kānuka nectar sample, was carried out by Stephen et al. [41]. The samples contained a total phenolic content of 424–1575 mg/kg and a methoxylated phenolic content of 64–665 mg/kg. Disparities were observed among the samples and were attributed to various amounts of other floral contributions, such as clover or mānuka. Kānuka honey was differentiated from mānuka by the ratios of some phenolic compounds, and it was suggested that the bioactivity of kānuka honeys is reliant on the variety and concentration of these compounds. A similar chemical profiling is also available in a study from Senanayake ([22], Chapter 7, Table 7.39), which was provided by Comvita New Zealand Ltd.

Kānuka nectar was reported not to contain dihydroxyacetone (DHA, the precursor of MGO), and thus resulted in an MGO-free honey [42]; however, Stephen et al. found traces of MGO in kānuka nectar and the resulting honey [41]. Stephen et al. also reported major phenolic components, such as those present in mānuka honey, with **gallic acid** and **4-methoxyphenyllactic acid** both at elevated levels and 2-methoxybenzoic acid as a minor fraction.

**Type II arabinogalactan proteins (AGPs)** were isolated from kānuka honey by Gannabathula et al. as well as apisimin [43], a honey-bee derived protein of unknown function that is usually present in royal jelly and has been reported to form a complex with apabulmin1 [44].

Kānuka and mānuka are from the same botanical family and tend to grow in similar environments. To offer pure monofloral honey to the consumer, a great deal of research has been carried out to find ways to discriminate mānuka honey from kānuka honey, based on various analyses. Therefore, the main research on the chemical profiling of kānuka honey has been performed in parallel to mānuka chemical profiling.

Stephen et al. showed evidence of mānuka and kānuka honeys sharing a common phenolic profile (**phenyllactic acid**, **methyl syringate**, a **methoxylated benzoic acid** and a structural isomer of **syringic acid**) [41]. While MGO was the main discriminant for mānuka honey, **phenyllactic acid** was a primary compound in kānuka honey, enabling distinction between the two floral sources. These results are supported by those obtained by Spiteri et al. through 1H NMR and chemometrics discrimination [30].

The fluorescence characteristics of various NZ honeys were investigated to establish if this technique might detect signatures unique to mānuka (n = 8) and kānuka honeys (n = 5) [33,45]. These studies identified two excitation-emission (ex-em) marker wavelengths each for mānuka and kānuka honeys (at 275–305 nm and 445–525 nm) that allowed for distinction between the floral types. The dilution of mānuka and kānuka honeys with other honey types that did not possess these fluorescence profiles resulted in a proportional reduction in the fluorescence signal of the honeys at the marker wavelengths. Thus far, only one of the fluorophores has been identified—**4-methoxyphenyllactic acid**, which is responsible for kānuka fluorescence at 275–305 nm. The other three are under investigation but are suspected not to be propolis-derived flavonoids [45].

In a study aiming to differentiate mānuka, kānuka and Australian jelly bush honey, Beitlich et al. identified kānuka honeys (n = 2 or 7) using volatile and non-volatile chromatography profiles [46]. Kānuka honey was characterized by **4-methoxyphenyllactic acid**, **methyl syringate**, **p-anisic acid** and **lumichrome**. Lumichrome is still a matter of debate, as it has been reported in other non-native honeys (Italian thistle and cornflower). A summary of potential kānuka markers is available in the report by Schmidt et al. [38].

#### 4.2.2. Biological Activity

##### Antibacterial Activity

Kānuka honey (n = 2) was tested by Molan for antibacterial activity against *S. aureus* [9]. It yielded the best inhibitory zone, better than mānuka honey (n = 12). However, it was acknowledged in the paper that the number of samples was not enough to draw reliable conclusions on the superiority of kānuka honey. The work was continued by Allen et al., whose results showed that kānuka honey (n = 20) displayed very high phenol-equivalent antibacterial activity against the same bacteria species, *S. aureus* (with a 90% confidence interval of 17.59–24.53% phenol (*w*/*v*)) [35]. Ninety-five percent of the kānuka samples had above average antibacterial activity, similar to rewarewa honey (n = 1) and better than mānuka (n = 50), kāmahi (n = 12) and rātā (n = 1) honeys. However, barberry honey (n = 3) was the most active honey tested in the study. The antibacterial activity of kānuka against *S. aureus* was greater in another study using the same method, where kānuka honey yielded a 27.9% phenol equivalent (*w*/*v*) [36], stronger than the previously assessed antibacterial activity in the report by Allen et al. [35]. In the same study, all the kānuka samples were active against *E. coli* at a minimum concentration of 19.3% (*v*/*v*), similar to buttercup and pennyroyal honey; however, mānuka remained the most active honey, keeping its antibacterial activity at a minimum concentration of 6.3% (*v*/*v*) [36].

According to a study by Lu et al., kānuka was less effective than mānuka and mānuka-kānuka blend honeys against *S. aureus*, *E. coli* and *Bacillus subtilis*, but was better than clover honey [37]. The two kānuka honeys had very low levels of MGO but displayed a non-peroxide antibacterial activity (assessed with catalase added to the honey). The authors observed a difference between the growth inhibition of *E. coli* and *B. subtilis* and the growth inhibition of *S. aureus*. They suggested that the chemical components required to inhibit growth are different for these species, and that the component which is specifically active against *S. aureus* could potentially only be activated by hydrogen peroxide (production or activation). In an experiment using *Pseudomonas aeruginosa*, kānuka honeys had similar effects to mānuka, inhibiting growth and displaying non-peroxide activity. Once again, the authors suggested the existence of non-MGO or non-peroxide component(s) preventing the growth of this bacterium.

Among all the native honeys tested by Allen et al., kānuka honey displayed some non-peroxide activity at very low levels (4.2% phenol equivalent vs. 15.5% for mānuka honey) [35]. The non-peroxide activity represented a very low proportion of the total antibacterial activity, contrary to mānuka honey. It was suggested that the non-peroxide activity was evidence of some mānuka contribution. Kānuka honey has not shown non-peroxidic activity in other studies, which could be explained by the absence of small amounts of DHA and MGO in kānuka nectar and honey, as discussed earlier [36,46].

In a randomised controlled treatment, kānuka honey was tested as a topical application for acne vs. an antibacterial soap. The medical-grade kānuka honey, Honevo©, was diluted with 10% glycerine and compared to a classic antibacterial facial soap. The results showed no evidence that the addition of kānuka honey was more effective than the use of antibacterial soap alone [47].

Braithwaite et al. suggested that the efficacy of medical-grade kānuka honey in the treatment of rosacea could be linked to its antibacterial activity against *Bacillus oleronius*, which is suspected to be involved in the inflammatory response [48].

##### Antifungal Activity

In another study, kānuka honey (n = 6) was tested for antifungal activity against *T. mentagrophytes* var. *mentagrophytes* in a modified agar well diffusion method. Five of the six samples tested were active; however, their antifungal activity was among the lowest, similar to that of rewarewa honey but lower than that of mānuka [36]. In the same study, kānuka honey favoured the growth of the yeast *C. albicans*.

##### Anti-Inflammatory and Immunostimulatory Activity

Leong et al. investigated native honeys, including kānuka honey (n = 4), for their anti-inflammatory effects. Kānuka honey samples exhibited a potent, dose-dependent reduction in human neutrophil superoxide production in vitro that was not correlated with the phenolic content of the said honeys [49]. The addition of MGO to low MGO-containing kānuka honey did not result in a higher anti-inflammatory effect, suggesting that something else was causing the suppression of neutrophil superoxide production. No free radicals were scavenged by mānuka honey, and it did not demonstrate any effect in vivo on artificially induced ear oedema compared to rewarewa and mānuka honeys.

According to a study by Gannabathula et al., kānuka honey shows higher immune stimulating effects than mānuka honey, and the active substances apparently disappear during storage [43]. The immunostimulatory activity of kānuka honey is mainly attributed to its **arabinogalactan proteins**, which originate from the nectar of the kānuka flowers. However, the immunostimulatory effect of AGPs at the concentrations present in kānuka honey was lower than the effect of whole kānuka honey, implying that other compounds in the honey might contribute to its activity. Apisimin, which stimulates the release of TNF-α from blood monocytes similar to AGPs, was also found in kānuka honey [44]. Apisimin displayed a synergetic action when combined with AGPs, which resulted in a three-fold release of TNF-α, but did not form a complex with the proteins.

In Bean’s thesis, the effect of different types of honey (concentration of 0.5%) on the phagocytosis of latex particles in LPS-activated THP-1 macrophages was studied. Among the 16 honey types, kānuka (n = 5) displayed the second highest percentage of inhibition of phagocytosis (mānuka excluded), with 15% inhibition (four times less active than mānuka honey). As phagocytosis produces large amounts of reactive oxygen species and ROS are pro-inflammatory and start the inflammatory cascade, it was hypothesised that the phagocytosis-inhibiting component(s) resulted in honey having anti-inflammatory activity. This could also be linked to macrophage polarisation from M1—in charge of pathogen killing—to M2, resolving the later stages of inflammation and tissue repair. The high molecular weight (HMW) fraction of honey was the active fraction during the assays [50].

In a study by Kuehne [51], kānuka honey demonstrated an anti-proliferative effect on macrophage RAW264.7 cells (LPS induced or not). Among the individual honeys, kānuka exhibited one of the highest anti-proliferative effects by reducing the proliferation rate by around 60% at the highest concentration tested (50 mg/mL). The treatment of the cells with kānuka honey resulted in a decreased production of NO—a reactive oxygen species involved in the immune response—at concentrations greater than 10 mg/mL. This result was observed in rewarewa honey as well, but at a higher concentration, meaning that kānuka honey keeps its antioxidant activity even when diluted. The comparison with artificial honey suggested that something other than just osmolarity was in play.

In the same study, kānuka honey presented pro-inflammatory activity by stimulating the production of tumour necrosis factor α (TNF-α)—a small cytokine released by macrophages as part of cell-signalling during the immune response. Combined, these results suggest that kānuka honey presents valuable immune-regulatory properties—it is pro-inflammatory, enabling it to deal with early stages of infection, and it is anti-inflammatory, enabling it to adjust the number of immune cells involved if they become too numerous and the ROS level too high [51].

To identify the chemicals involved, the honeys were separated into different fractions: high molecular weight (HMW) and low molecular weight (LMW). It was then found that the HMW fraction had the pro-inflammatory properties, whereas the LMW—excluding the hydrophobic phenolic compounds and mono- and disaccharides—was found to be responsible for the inhibition of NADPH oxidase, which is a membrane-bound enzyme present in neutrophils that is responsible for the production of ROS after a respiratory burst. The extraction methods and mechanisms of action were discussed by Kuehne [51] (Chapter 7, § 7.2 and § 7.3) and were linked to a reduction in the membrane translocation of Rac2—a G protein in charge of activating the dormant NADPH oxidase.

According to a study by Tomblin et al., one of the potential anti-inflammatory pathways may be related to Toll-like receptors (TLRs)—proteins that play a key role in the innate immune system by recognizing structurally conserved molecules derived from microbes [52]. The authors worked on three different cell lines, all targeting a different chemical pathway. Their results showed that treatment with kānuka honey resulted in powerful anti-inflammatory effects in HEK-Blue™-2 cells, but not in HEK-Blue™-4 or NOD2-WT cells. Specifically, the anti-inflammatory effect occurred via the TLR1/TLR2 signalling pathway, which is consistent with Gannabathula’s results [44]. In this experiment, they tested crude honey and phenolic extracts, with the results showing that a higher phenolic content produced an elevated anti-inflammatory effect. However, subsequent investigation is needed to determine the specific compounds present in kānuka honey that are responsible for its anti-inflammatory activity.

A randomized controlled trial with 15 participants was undertaken to assess the efficacy of medical-grade kānuka honey for the treatment of eczema [53]. The cause of eczema is still under investigation; however, the current proposed explanation is that the immune system overreacts and causes inflammation and other typical symptoms (itchiness, redness, flaky skin, etc.). In this pilot single-blind randomised controlled trial, the topical application of kānuka did not appear to be effective in the management of eczema. The authors suggested that such a study should be conducted with a larger group of patients.

In another study, the anti-inflammatory and immunoregulatory action of kānuka honey was investigated for the treatment of psoriasis. This chronic autoimmune condition causes a rapid build-up of skin cells, which is associated with localised inflammation. Fingleton et al. performed a randomised controlled trial with 15 participants that compared medical-grade kānuka honey and an aqueous cream, one of the recommended treatments. Honey appeared to have similar effects to the aqueous cream but was still less efficient than the steroid topical application. The use of steroids in the long run is not recommended, justifying the interest in honey as an alternative to deal with mild symptoms. Here again, the main limitation was the small sample size, and the authors recommended a confirmation of their conclusion with a “suitably powered study” [54].

In another study, Braithwaite et al. investigated the use of medical-grade kānuka honey for the treatment of rosacea, a common chronic inflammatory skin condition. In their 138-patient randomised controlled trial, Honevo (90% honey, 10% glycerine) was compared to cetomacrogol (a paraffin-based topical emollient). The results showed that kānuka honey was an effective and well-tolerated treatment [48]. However, the authors acknowledged some limitations: firstly, the odour of Honevo, which made blind tests impossible; and secondly, the difficulty of assessing the severity of rosacea (inherent varied clinical characteristics) and its evolution with treatments. The mechanism of action was not assessed in this study; however, the authors discussed the potential for such further research to be performed based on the literature discussed earlier in this section, including that on antibacterial properties.

Medical-grade kānuka honey was also used to treat actinic keratoses, common skin lesions that form as rough, scaly plaques of slow-growing epidermal keratinocyte dysplasia. These lesions are usually present in the elderly as a result of chronic and cumulative sun exposure. Mane et al. reported one successful treatment with kānuka honey and discussed the implications of the immunoregulatory effects of the arabinogalactan proteins previously reported in kānuka honey [55].

##### Antiviral Activity

Kānuka honey was investigated by Fingleton et al. as an alternative medical treatment for cold sores, which are blisters on the lips resulting from the herpes simplex labialis virus. In this randomised controlled trial (n = 15), kānuka honey appeared to be an acceptable treatment; however, the authors acknowledged the small number of patients involved in the trial and the impact it had on the reliability of the significance of the results [56]. This work was continued by Semprini et al. with the comparison of medical-grade kānuka honey and aciclovir (an antiviral topical treatment) for the treatment of the same symptoms. This study involved 956 patients; the results of the randomised controlled trial showed no difference in efficacy between honey and the classic antiviral topical treatment. Therefore, kānuka honey offers the possibility of being used as an alternative medical treatment method, especially in the case of allergies or resistance to drugs [57].

#### 4.2.3. Summary and Research Gaps

Among all the honeys presented in this review, the amount of literature available on kānuka honey is comparatively abundant, mainly due to its resemblance to mānuka honey and the common mānuka/kānuka honey blend that is present on the market. The identified chemical characteristics are as follows: the presence of aromatic compounds; different ratios of phenolic compounds; and the floral markers **4-methoxyphenyllactic acid**, **methyl syringate**, **p-anisic acid**, and **lumichrome.** Fluorescence analysis was able to identify **4-methoxyphenyllactic acid** at ex-em 275–305 nm; however, the second peak at ex-em 445–525 nm has yet to be identified. Combined, they could justify the use of fluorescence as a non-destructive and rapid screening method to identify kānuka honey.

Due to its previously reported bioactivity, kānuka is available on the market as a medical-grade honey (Honevo©). Medical-grade kānuka honey has been shown to have immunomodulatory and anti-inflammatory effects in vitro and is emerging as a viable and well-tolerated treatment for dermatological lesions. Braithwait et al. proposed causes for rosacea and reasons why kānuka honey was effective; they mentioned the potential implication of *Demodex folliculorum*, a type of mite that lives in human hair follicles and feeds on dead cells [48]. Thus far, to our knowledge, no study has been undertaken to investigate the effect of honey on problematic microscopic species that are not bacteria, yeasts or fungi.

### 4.3. Northern Rātā

#### 4.3.1. Properties and Floral Markers

After consulting the literature available on rātā honey, it is evident that northern rātā (*Metrosideros robusta*) is poorly studied. The main results presented in the following paragraphs generally refer to *M. umbellata*, or southern rātā.

A mineral analysis of southern rātā honey (*M. umbellata*) was carried out by Vanhanen et al. [21]. The colour, pH, moisture, conductivity and mineral content of the honey was compared to other NZ native honeys. Rātā honey is a very light-coloured honey, with an average moisture content of 18 g per 100 g fresh weight, a pH just below 4.0 and a conductivity of 0.6 mS/cm. The main minerals present are potassium, sodium, sulphur and calcium, with a mean total mineral content slightly above 1000 mg/kg [21]. Langford et al. also analysed colour and moisture (consistent with the previously mentioned study), but added the measurement of HMF, fructose and glucose contents. It was shown that rātā honey was the highest carbohydrate-containing honey in the study [23].

A first list of extractives for *Metrosideros* spp. is included in Hyink’s work. There was no distinction between northern/southern/vine rātā and the author acknowledged the high probability of the samples being multifloral [25]. Goss’s thesis includes a GC-MS profile and the peak identification of methylated rātā honey (*M. umbellata*), including the corresponding compound concentrations (mg/kg) [24]. Rātā honey was found to contain low levels of extractable organic substances (typically >50 mg/kg).

Using a list of 22 compounds previously identified by SIFT-MS, Langford et al. performed two PCAs. The second, with **dimethyl sulphide** as the highest discriminative compound, allowed the authors to cluster rātā honeys together as a distinctive group from other NZ honeys [23].

Langford et al. used selected ion flow tube-mass spectrometry to characterize monofloral NZ honeys, including rātā, based on their aroma signature. After the analysis, they were able to list 22 compounds and their concentrations (in μg/L) that were present in the samples. They performed two multivariate analyses on their data and successfully clustered the honeys by botanical origin. They stated that “the most effective were the compounds with the highest volatility: methanol, ethanol and acetaldehyde”. They also noted that these compounds could be considered less reliable in postharvest analysis due to the fermentation that occurs in honey. After removing those volatile compounds, **dimethyl sulphide** yielded the highest discriminatory power and allowed them to distinguish rātā honey from the other honeys. The authors acknowledged that the samples could be variable between harvesting seasons; however, they were confident in the reliability of their results and in the use of **dimethyl sulphide** for quality assurance [23].

Goss [24] attempted to determine the extractable organic substances of understudied native honeys, including rātā (*M. umbellata*). The method used was near infrared spectroscopy (scanning for 8000–3850 cm^−1^, statistical analysis performed on a sub-dataset in the spectra range of 6000–3850 cm^−1^) to identify indicators of floral origin. The statistical analysis, which created clusters based on the identified components, successfully segregated rātā honey from other floral sources. The study offers a flowchart of the multistep determination of floral source using NIR classification and conductivity, colour, sugar and pollen analysis [24] (Chapter 6, § 6.4.1).

Another flowchart was presented by Petchell [32] (Chapter 3, § 3.5), which classified rātā honey by the combination of the absence of several compounds and the presence of **dimethyl sulphide** and **dimethyl sulfoxide. Dimethyl sulphide** and **dimethyl sulfoxide** were the two compounds used to discriminate rātā (*M. umbellata*) honey in Petchell’s work; however, they have been reported to be present in other honey types (rosemary, orange, eucalyptus, avocado, thyme and oak, as cited in [32] and [23]) from various locations (Spain, Lithuania and NZ).

Bong et al. analysed various honeys, including *M. umbellata* (n = 3), using a chemical and fluorescence profiling approach. The multivariate analysis failed to cluster rātā samples in a distinctive group, showing that the method and the chemicals analysed in the study were not suitable for rātā profiling [45].

Rātā honey typically contains a proportion of kāmahi honey, as both species grow in the same area with an overlapping flowering period. The consistent contribution of kāmahi as a secondary nectar source in rātā honey can be confirmed by pollen analysis. It is also common for rātā honey to contain **meliracemoic acid** (<2.5 mg/kg) or **kamahines** (<6 mg/kg), which are floral markers for kāmahi honey [24].

In the study by Bong et al., the chromatograms of rātā and pōhutukawa (n = 3) were almost identical. Knowing the high frequency of hybrids, it may be safe to suggest that the chosen wavelength or method itself was not suitable to differentiate the two species [45]. Pōhutukawa honey can be identified by other compounds (**3-methylbut-2-enal** and **(E)-cinnamaldehyde**) that are not found in rātā honey [32]. The chromatograms of tāwari and clover were also analogous at the chosen wavelength in the study by Bong et al. [45], reinforcing the conclusion that this may not be an appropriate approach to distinguish rātā honey from other floral sources.

#### 4.3.2. Biological Activity

##### Antibacterial Activity

Molan et al. included rātā and white rātā in their study on the antibacterial activity of NZ honeys against *S. aureus* (ATCC 25923) [34]. It is unclear how many samples were used, as the results only mentioned the common name “rātā” with n = 2. The activity was measured as the inhibitory zone in an agar diffusion assay, and the mean was weighted to allow a fair comparison between floral sources. Rātā yielded one of the smallest inhibitory zones, with only 0.15 mm^2^ vs. 14.64 mm^2^ (n = 12) for mānuka (*p* < 0.0001).

In another study, Allen et al. tested the antibacterial activity of different rātā species, including white rātā (*M. perforātā*), rātā (*M. robusta*) and vine rātā (*M. fulgens*), using phenol equivalents in an agar diffusion assay (*S. aureus* (ATCC 25923)). Vine rātā and rātā had some of the lowest observed antibacterial activities; both species were below the level of detection in the assay (n = 2, n = 2). White rātā yielded a better score (8.65%, n = 2); however, it was still lower than the median activity of the 345 native honeys tested (13.6%). It is worth noting that rātā honeys were among those with the lowest number of samples tested (n = 2) compared to mānuka (n = 50). None of the rātā species were reported to have any non-peroxide antibacterial properties [35].

The antibacterial activity of non-mānuka honeys against *E*. *coli* and *S. aureus* was studied by Brady et al. (2004). Their rātā honeys included white rātā (n = 2), rātā (n = 6) and another species from the *Metrosideros* family, pōhutukawa (*Metrosideros excelsa*, n = 8). Against *S. aureus*, three out of six samples of rātā yielded an activity of 11.4%, which was lower than the white rātā samples (13.4%) but higher than the one pōhutukawa sample (8.2%). All three species were less active than kānuka, rewarewa or mānuka honeys, but had similar activity to kāmahi honey. The results against *E. coli* showed different antibacterial activities, measured as minimum inhibitory concentration (% honey *v*/*v*), including two active white rātā (23.4%) and two active pōhutukawa (23.4%) samples; none of the two rātā samples were active against *E. coli*. While mānuka remained active at a concentration of 6.3%, white rātā lost its bioactivity after a certain level of dilution. Overall, rātā honey did not show any non-peroxide activity. In this study, the number of samples for *Metrosideros* spp. was very low compared to other types (e.g., n = 18 for honeydew honey) [36].

##### Antifungal Activity

Brady et al. also included an antifungal assay with rātā honeys; however, neither of the two rātā species were active against *T. mentagrophytes* var. *mentagrophytes* or *C. albicans* (which was enhanced by the sugar content). Five pōhutukawa honeys were detected as being active against *T. mentagrophytes* var. *mentagrophytes*, showing evidence that *Metrosideros* spp. do not share the same antifungal properties/components [36].

#### 4.3.3. Concluding Remarks and Research Gaps

As stated at the start of this section, the scarce literature on rātā seems to have a strong focus on southern rātā (*M. umbellata*). Firstly, in future research, it would be interesting to assess whether the results obtained in these studies are also applicable to *M. robusta*. In terms of chemical composition and floral markers, **dimethyl sulphide** and **dimethyl sulfoxide** seem to be the factors that differentiate *M. umbellata* from other honeys. The questions remain: Is there a way to differentiate northern and southern rātā honey using these markers? Are other markers necessary? Is it of major importance for the industry (quality assurance, labelling, etc.) or is it irrelevant at this stage? It would be interesting to see if those sulfuric compounds are related to the soil and whether they are also present in other plants growing alongside rātās.

Regarding the bioactivity of rātā honey, the three studies that included rātā honey in their experiments did not focus on that honey type, as attested by the small number of samples. Overall, rātā honey does not seem to have a particularly strong antibacterial or antifungal activity; however, one should remember that only a few bacteria, yeast and fungi species were tested. Because of this unpromising bioactivity, no study has reported an assessment of antioxidant, immune-regulatory, anti-inflammatory or antiviral activities for rātā honeys.

### 4.4. Rewarewa Honey

#### 4.4.1. Properties and Floral Markers

Rewarewa honey is a dark-coloured honey with a pH of 4.21, a conductivity of 0.61 mS/cm and a moisture content of 16.5 g per 100 g of fresh weight. Its mean total mineral content is 1548 mg/kg, with potassium as the main mineral component (1290 mg/kg) [21]. These measurements are consistent with those of Langford et al., which were determined using the HMF content and carbohydrate contents (35.3% fructose, 29.1% glucose) [23].

Rewarewa cannot be identified by pollen analysis as the flowers are pollinated by nectar-feeding birds: bees can collect nectar from the flowers without dislodging pollen from the anthers [9,21,23]. Therefore, the identification of rewarewa honey must rely on other criteria.

Parts of the extracted compounds from rewarewa honey (n = 4) were presented in a study by Wilkins and Lu, which identified eighteen aliphatic dicarboxylic acids in methylated extracts by GC-MS analyses. While **butanedioic acid**, **decanedioic acid** and **2-decenedioic acid** are dominant rewarewa constituents, they also occur in many other monofloral New Zealand honeys; hence, their detection does not assist in floral source discrimination. The total aliphatic diacid content was proposed as a defining characteristic, rather than the concentration of individual compounds [56].

An analysis of the phenolic compounds and MGO in some NZ honeys was carried out by Stephen et al., who included only one sample of rewarewa. The overall results showed a low phenolic content compared to mānuka and kānuka honeys, an absence of MGO and a relative abundance of phenyllactic acid and trimethoxybenzoic acid compared to the other compounds. They suggested using the low phenolic composition as a discriminant for diluted mānuka and kānuka honeys [41].

After the examination of more than 200 NZ honey samples, **2-methoxybutanedioic acid** and **4-hydroxy-3-methyl-*trans*-2-pentenedioic acid** were detected only in samples possessing a significant rewarewa contribution, hence their importance in the characterization of the floral source [56]. These markers were used in other later studies [22,32], and the threshold levels proposed were 2.3–3.3 mg/kg for **2-methyoxybutanedioic acid** and 0.2–3.9 mg/kg for **4-hydroxy-3-methyl-*trans*-2-pentenedioic acid** [24]. The high content of aliphatic diacids was also proposed as a discriminant floral marker in the range of 64–111 mg/kg [24].

Using probability plots (see earlier), all the honeys from the same floral source were analysed together and then removed from the data, leaving rewarewa as the last floral source to be studied in Petchell’s work. As the remaining monofloral honey type, it was not possible to classify rewarewa honey by means of the presence or absence of specific compounds, as there was nothing to compare it with and because rewarewa did not exhibit any defining chemical features from the chosen list of compounds [32].

Using NIR spectroscopy, Goss successfully segregated rewarewa honey from other floral sources [24]. The study offers a flowchart of a multistep determination of floral source using NIR classification and conductivity, colour, sugar and pollen analysis [24] (Chapter 6, § 6.4.1). In the flowchart, rewarewa honey is classified as a “light-coloured honey” compared to “dark-coloured” clover and thyme honeys, which disagrees with previous studies. This is most likely a typo; however, it is important enough to notice, as it could compromise the reliability of the flowchart itself.

The PCA performed on the concentration of various compounds (assessed by application of selected ion tube-mass spectrometry) of native honeys, including rewarewa (n = 5), failed to group the rewarewa samples as a distinct cluster once methanol and ethanol were removed from the discriminating compounds [23].

In a preliminary study on the fluorescence of native honeys, Bong et al. noticed a lack of fluorescence from rewarewa honey at the wavelengths chosen to target mānuka honey markers [33]. This lack of fluorescence was of importance to identify the contribution of non-mānuka honey and the dilution that resulted, which was noticeable as a decreased fluorescence. Bong et al. then continued to analyse various honeys, including rewarewa (n = 6), using a chemical and fluorescence profiling approach. The chromatogram (at 265 nm) appeared to be far less complex than other honeys (mānuka, kānuka or NZ ling) [45]. While rewarewa honey is known to be rich in aliphatic dicarboxylic acids [56], the authors were unable to detect these compounds in this study. They suggested that a derivatisation procedure is most likely necessary for the quantification of these compounds.

When MGO, dihydroxyacetone and leptosperin are used as discriminants, combined with 1H NMR data, rewarewa can only be classified as a non-mānuka honey and is grouped with kānuka and other native honeys [30].

#### 4.4.2. Biological Activity

##### Antibacterial Activity

Molan et al. (1988) tested the antibacterial activity of NZ honeys against *S. aureus* (ATCC 25923) with four samples of rewarewa honey. The activity was measured as the inhibitory zone in an agar diffusion assay; the mean was weighted to allow for a fair comparison between floral sources. Rewarewa activity displayed an inhibitory zone of 1.32 mm^2^, which was less than that of kānuka and kāmahi but greater than that of rātā honeys [40].

In the study by Allen et al., the antibacterial activity of native honeys was assessed using phenol equivalents in an agar diffusion assay (*S. aureus* (ATCC 25923)). Only one rewarewa sample was included in the experiment and yielded an activity greater than the 345 honeys’ average activity (20.9% vs. 13.6%). Its activity was among the highest, along with barberry (41.4%), pennyroyal (25.3%), oilseed rape (22.0%) and kānuka (21.95%). The one sample of rewarewa displayed a greater activity than the mānuka honey average (15.45%, n = 50). Interestingly, pasture honey (mixed sources) showed an activity greater than rewarewa honey, with 21.4% (n = 3) [35].

According to Brady et al., rewarewa did not display any non-peroxide activity against *S. aureus*; however, it yielded an antibacterial activity average of 16.9% (n = 22, 100% of the samples were active), similar to eucalyptus honey, greater than rātā and lower than the mānuka standard (28.4%) and kānuka (27.9%, n = 6). In the same study, 17 samples of rewarewa honey were shown to be active against *E. coli* with a mean minimum inhibitory concentration of 22.0% (*v*/*v*), which was slightly better than the vast majority of the honeys’ MICs (23.4%) but worse than that of mānuka (6.3%) [36].

Rewarewa honey has been shown to possess antibacterial activity against *Pseudomonas mirabilis*, *E. coli*, *Proteus mirabilis*, *Salmonella typhimurium*, *S. aureus* and *Streptococcus pyogenes* [57]. In this study, the activity ranged from 10.5% (*w*/*w*) against *E. coli* to 4.0% (*w*/*w*) against *S. pyogenes*. This honey was compared to a known mānuka honey, and the results showed that the bacteria displayed various levels of sensitivity to honeys from different floral sources, even if the statistical analysis of the median response value showed no significant difference in the overall level of activity between the two honey types. *E. coli* and *S. aureus* were clearly much more susceptible to mānuka honey. *P. mirabilis* and *S. pyogenes* were more susceptible to rewarewa honey. The other species tested had a similar susceptibility to both honey types.

Wilkinson and Cavanagh continued the work on the antibacterial activity of various honeys against *E. coli* and *Pseudomonas aeruginosa.* All honey tested, including rewarewa, displayed a statistically significant antibacterial activity against *E. coli* at 10% (*w*/*v*) and 5% (*w*/*v*). Rewarewa honey (10%) produced the greatest inhibition of *E. coli*, with the zones of inhibition being significantly larger than those of mānuka, in contrast to the results of Willix et al. (1992). At 2.5%, rewarewa honey displayed antibacterial activity; however, it was not statistically different from the other honeys tested. The same experiment was performed with *P. aeruginosa*, another major wound-infecting bacteria, and rewarewa did not show a statistically significant antibacterial activity at any concentration level tested. No non-peroxide activity was detected for rewarewa honey in the study. A phenol equivalence was calculated for all honeys from the phenol standard curve and the phenol equivalence for rewarewa, the best honey against *E. coli*, was 54% [58].

According to the study by Bukahri (2014), rewarewa honey displayed one of the highest antibacterial activities against *S. aureus* and *P. aeruginosa* when compared to mānuka and other international honeys in vitro in agar diffusion assays. Included in this PhD thesis is a comparison of the minimum inhibitory concentration (MIC) and minimum bactericidal concentration (MBC) of some international honeys. Against *S. aureus*, rewarewa honey needed a 10% concentration to inhibit bacterial growth in a bacterial suspension but a 20% concentration to become bactericidal (same bacterial suspensions tested for MIC inoculated on agar plate; MBC defined as the minimum concentration that resulted in no growth on the agar plate). These concentrations were similar to mānuka honey, but not as ideal as those of Chilean rainforest honey. Rewarewa honey was not affected by catalase and retained its activity against *S. aureus*, indicating the involvement of complex antibacterial agents in addition to a small amount of hydrogen peroxide. *Fusobacterium nucleatum*, an anaerobic wound-infecting bacteria species, was used to assess the antibacterial activity of the same international honeys. Mānuka yielded the highest activity when highly concentrated (100–80%), while rewarewa displayed a better antibacterial activity when diluted (40–20%). At 10% concentration, all honeys tested had the same effect on *F. nucleatum* [59].

In the same study, Bukhari investigated the effect of honey on *S. aureus* and *P. aeruginosa* biofilms—aggregations of bacteria cells in an elaborate structure enclosed within a self-produced extracellular polymeric matrix. The concentration of antibiotics required to attack a biofilm was reported to be about 100 to 1000 times greater than the concentration needed to kill the same bacteria in the free-swimming form [59]. All honeys tested, including rewarewa, completely inhibited bacterial biofilms at concentrations above 20 mg/mL. As the author said, if honeys are “applied directly to the wound and therefore the associated biofilms, concentrations of this order will inevitably be achieved, when raw honeys are applied”, offering a desirable antibiotic alternative to deal with biofilms. Preliminary work is also available in the thesis on the effect of honey on biofilm formation in response to quorum sensing and its effect on toxin production (killing the pathogen vs. blocking the toxin production by the pathogen).

##### Antifungal Activity

In one of Brady et al.’s studies, *T. mentagrophytes* var. *mentagrophytes* was shown to be susceptible to rewarewa honey, where 13 samples were active (mean zone of inhibition of 12.8 mm). It was one of the 13 floral sources out of 27 to display antifungal activity. As previously stated, *C. albicans* was enhanced by the sugar content of all honeys [36].

##### Anti-Inflammatory, Antioxidant and Immunostimulatory Activity

Leong et al. studied 21 indigenous NZ honeys for their anti-inflammatory activity based on the previously reported activity of phenolic compounds in honey. The authors tested if honey could suppress the production of superoxide by human neutrophils in vitro. One of the samples was a rewarewa honey, displaying no MGO. The neutrophil superoxide inhibition (IC_50_) of this rewarewa honey was 4.3 mg/mL, which was among the best activities, similar to kānuka (n = 4) and better than mānuka (n = 15). No correlation was found between the superoxide inhibition and phenolic content, contrary to what the authors initially hypothesized. A significant inverse correlation was found between anti-inflammatory activity and MGO content; however, further experiments showed that it was not caused by the MGO-dependent inhibition of anti-inflammatory activity. No cell death was reported; however, at a higher concentration than its IC_50_, rewarewa caused cell death in vitro, as did other tested honeys. In a cellular free radical scavenging assay, none of the honeys displayed ‘ROS scavenging’ activity. Together, these results indicated that the anti-inflammatory activity of the rewarewa honey was likely caused by the inhibition of the neutrophil respiratory burst [49].

Leong et al. further tested rewarewa honey on artificially induced ear oedema in mice (topical inflammation in vivo). Out of the three most effective anti-inflammatory honeys previously identified in vitro, only rewarewa significantly reduced AA-induced oedema compared with the untreated negative control group; this effect was similar to that of the positive control, dexamethasone. This result indicates that rewarewa honey has the potential to abrogate inflammation by hitting multiple inflammatory targets, including the neutrophil respiratory burst, neutrophil recruitment and swelling. However, more rewarewa honey samples from different regions in NZ need to be tested before any claims can be made about the superiority of honey of rewarewa origin with respect to anti-inflammatory activity [49].

The effect of different types of honey (concentration of 0.5%) on the phagocytosis of latex particles in LPS-activated THP-1 macrophages was studied by Bean. Among the 16 honey types, rewarewa (n = 3) displayed the highest percentage of inhibition of phagocytosis (mānuka excluded), with 20% inhibition (similar to the three least active mānuka honeys at the same concentration). Rewarewa was found to have no significant effect on ROS release and displayed no significant ROS scavenging activity (ROS released by actively phagocytising macrophages) [50].

These results were later confirmed by Kuehne (2016). In this study, rewarewa honey exhibited the highest anti-inflammatory activity as a high inhibitory activity on the production of superoxide by PMA-stimulated neutrophils. It was suggested that rewarewa honey negatively influences the functioning of NADPH oxidase, a membrane-bound enzyme present in neutrophils that is responsible for ROS production after a respiratory burst. Preliminary experiments have been carried out to identify the molecule(s) interacting with NADPH oxidase in rewarewa honey—not phenolic, but potentially peptides, amino acids or organic acids [51]. **Succinic** and **azelaic acids** have both been reported to inhibit neutrophil respiratory bursts in vitro and have been found in rewarewa honey [56,60]. The extraction methods and mechanisms of action were discussed by Kuehne [51] (Chapter 7, § 7.2 and § 7.3).

Kuehne also showed the immune-regulatory effects of rewarewa honey using LPS-stimulated macrophages (cell line RAW264.7) in vitro [51]. The addition of rewarewa honey, in the presence of LPS, stimulated the proliferation of macrophages up to a limit concentration, when honey started reducing proliferation in a dose-dependent manner. In the absence of LPS, all honey types decreased the proliferation of macrophages. Rewarewa also reduced the production of NO by the LPS-stimulated macrophages, but only at a high concentration—NO is a potent inflammatory mediator released from activated immune cells. Without LPS, the HMW fraction of rewarewa honey induced the production of NO, as well as regulating compounds such as TNF-α and IL-6, up to a certain concentration, where the anti-proliferative effect became the main inflammation-modulatory mechanism. To the contrary, the LMW fractions of rewarewa honey showed an inhibitory effect on the production of NO and IL-6 in LPS-stimulated RAW264.7 cells, which was mainly due to its anti-proliferative effect [51].

##### Antiviral Activity

Littlejohn studied the sensitivity of *Adenovirus* and *Herpes simplex virus* (HSV) to different honeys, including rewarewa, with its proven antioxidant activity. Using a variety of cell cultures, the sensitivity of the viruses was assessed by monitoring morphological changes to the cells. The study aimed to understand the effect of honey, including protection, i.e., testing if honey-treated cells would be protected from the virus and infection; prevention, i.e., testing if honey-treated infected cells would prevent the propagation of the virus to healthy cells; and neutralization, i.e., assessing the impact of the direct exposure of honey to the viruses [61].

Rewarewa honey displayed an effect on HSV-1 in the neutralisation assay at a 10% (*v*/*v*) concentration for a 4h treatment, and at 5% and 10% concentrations for an 8 h treatment. It was suggested that rewarewa honey has a virustatic rather than a virucidal activity, due to the development of symptoms of cytopathogenic effect—structural changes in host cells that are caused by viral invasion—in another assay with the same concentrations and treatment time [61].

#### 4.4.3. Summary and Research Gaps

The chemical profiling of rewarewa honey is still incomplete. The data available focus on its mineral, aliphatic diacids and phenolic compounds. Rewarewa honey is free of MGO and displays chemical characteristics that allow its discrimination from other honey types. Among them, three floral markers have been identified: **high aliphatic diacids content**, **2- methoxybutanedioic acid** and **4-hydroxy-3-methyl-trans-2-pentenedioic acid**.

The bioactivity of rewarewa honey has been studied in various bacteria species but only one fungus. It is among the most antibacterial native honeys; however, it does not display non-peroxide activity, with the chemical analysis showing a lack of MGO (typically associated with this activity). The most commonly studied topic is the anti-inflammatory activity of rewarewa honey, which is linked with its antioxidant and immune-modulatory effects. It seems that rewarewa honey has an impact on the respiratory burst, not as a free ROS scavenger, but rather directly affecting the production of reactive species involved in the immune reaction. No molecules have been clearly identified yet, but there are enough suggested leads to continue the investigation. It would be valuable to further investigate **succinic** and **azelaic acids** in rewarewa honey, as they are suspected to be involved in its immune-regulatory activity. Finally, its antioxidant activity could be linked to the potential antiviral effect of rewarewa honey against Herpes simplex virus 1.

## 5. Conclusions

With increased mānuka honey production putting pressure on the land and monopolising consumer interest, it is time to explore the viability of other non-mānuka monofloral native honeys. Diversifying the market could lead to a more sustainable income for beekeepers, reducing pressure on Māori beekeepers for whom non-mānuka honey production is not currently profitable, which contributes to the under-utilisation and investment in conservation land while also taking the opportunity to promote native flora and Māori values in land management decision making. If new biologically active constituents in honeys from NZ native plants are identified as being beneficial for a wider range of human and animal health problems, then the honey industry could benefit from diversification, which would also reduce the uncertainties experienced by producers relying on mānuka honey alone.

This review summarized the existing knowledge on four native plants and their honeys and identified the research gaps that, once answered, will allow informed and science-led marketing decisions to be made. Table 2 provides an overview of the key compounds and biological activities for the four native monofloral honeys investigated.

From the study of the available published literature and publicly available data about honey as a therapeutical alternative, there is no denying that the medical field could benefit from additional research on various honey types and their biological properties. Many published reports acknowledge honey as a viable alternative to pharmacological treatment for various illnesses and conditions. Pharmacological treatments can be effective; however, they often come with undesired side effects. The use of honey could offer an alternative to reduce such side effects.

In addition, a better understanding of the rongoā (traditional medicine) of native plants and their honeys could add the spiritual dimension, taha wairua, that is missing in synthetic products and Western medicine. Bringing to light the science-based evidence that supports honey produced according to Māori values could provide health care for people who do not trust Western medicine or cannot afford it. The rising interest in traditional medicine nationally and internationally is also an opportunity to promote Māori values in NZ and overseas, while contributing to NZ’s economy and Māori aspirations for intergenerational holistic wellbeing.

Some interesting properties were highlighted is this report, with kānuka and rewarewa being the most promising alternative honeys. Despite the volume of research investigating their antibacterial activity, it would be beneficial to increase the number of samples in future studies to allow for reliable statistical analysis, increase the number of bacterial species studied and to perform more in vivo studies. Antifungal activity remains largely understudied for NZ native honeys, with only one fungus species being explored thus far. Rewarewa and kānuka honeys displayed antiviral properties; however, in vitro and in vivo clinical trials need to be undertaken to provide evidence that is strong enough to suggest honey as a viable alternative.

The link between specific chemical components, the mechanisms of action and clinical efficiency is yet to be investigated. First, it would be interesting to assess the concentration of bee-defensin-1, apalbumin and apisimin in all native honeys. Then, studies should be undertaken to identify the molecules involved, e.g., arabinogalactan proteins in kānuka or succinic and azelaic acids in rewarewa.

Quality assurance is another important aspect moving forward. Floral markers of monoflorality are available for the four native honeys of interest; however, it is crucial to consider the impacts of monofloral honey production on conservation land and local economies. It would be worthwhile to include blends of various native flowers in future studies to assess if the combined properties are complementary, synergistic or cancel each other out. The question of geographical origin also needs to be considered, as environmental factors, such as climate, rainfall and surrounding vegetation, are known to affect the quality of honey and other bee products [62,63].

Overall, further work to gain a better understanding of the biochemical and medicinal properties of non-mānuka monofloral honeys could bring the impetus that Aotearoa New Zealand needs for its monofloral native honeys to be competitive in the national and international markets, promoting Māori vision, aspirations and values and developing cost-effective medical alternatives while investing in and protecting its unique natural resources.

## Figures and Tables

**Table 1:** Uses of rewarewa, kānuka, mānuka, northern rātā and kāmahi in rongoā (traditional Māori medicine) [16].

		*K. excelsa* (Rewarewa)	*K. ericoides* (Kānuka)	*L. scoparium* (Mānuka)	*M. robusta* (Northern Rātā)	*W. racemosa* (Kāmahi)
MEDICINAL USES	Wound care	Bark		Bark, fruit and other plant parts	Bark, leaves and sap	Bark
	Cold and flu-like symptoms			Bark, leaves and other plant parts	Bark and nectar	Other plant parts
	Digestive afflictions		Fruit	Bark, leaves, fruit and other plant parts	Bark	
	Urinary and genital afflictions			Bark and leaves	Bark	
	Skin afflictions			Bark	Bark	
	Dental care		Bark	Bark and sap	Leaves	
	Pain relief				Bark	
	Others	Bark	Bark and leaves	Bark and leaves		

**Table 2:** Summary of chemicals and biological activities of the four honeys of interest investigated in the literature. This table does not display a proven activity but rather the work that has been carried out so far. For more details, please read the corresponding sections of this report.

Plant Species	Rātā (*M. umbellata*)	Kāmahi (*W. racemosa*)	Kānuka (*K. ericoides*)	Rewarewa (*K. excelsa*)
Noteworthy chemicals (floral markers or active compounds)	dimethyl sulfide dimethyl sulfoxide	kamahines A, B and C meliracemoic acid 2,6-dimethylocta-3,7-diene-2,6- diol 3,3,5-trimethylcyclohex-2-ene-1,4-dione 2,2,6-trimethylcyclohexane-1,4-dione cis,trans-abscisic acid trans,trans-abscisic acid	4-methoxyphenyllactic acid type II arabinogalactan proteins methyl syringate p-anisic acid lumichrome	high aliphatic diacids content 2- methoxybutanedioic acid 4-hydroxy-3-methyl-trans-2-pentenedioic acid succinic acid azelaic acid
Investigated bacteria species and related afflictions	*Escherichia coli* *Staphylococcus aureus*	*Escherichia coli* *Staphylococcus aureus* Kamahines bioactivity assays *: *Escherichia coli* *Staphylococcus aureus* *Pseudomonas aeruginosa* *Bacillus subtilis*	*Escherichia coli* *Staphylococcus aureus* *Pseudomonas aeruginosa* *Bacillus subtilis* Afflictions: Acne Rosacea	*Escherichia coli* *Staphylococcus aureus* *Staphylococcus aureus* (biofilm) *Pseudomonas mirabilis* *Proteus mirabilis* *Salmonella typhimurium* *Streptococcus pyogenes* *Pseudomonas aeruginosa* *Pseudomonas aeruginosa* (biofilm) *Fusobacterium nucleatum*
Investigated fungi species and related afflictions	*Candida albicans* *Trichophyton mentagrophytes var. mentagrophytes*	*Candida albicans* *Trichophyton mentagrophytes var. mentagrophytes* Kamahines bioactivity assays *: *Candida albicans* *Trichophyton mentagrophytes* *Cladosporium resinae*	*Candida albicans* *Trichophyton mentagrophytes var. mentagrophytes*	*Candida albicans* *Trichophyton mentagrophytes var. mentagrophytes*
Investigated viruses and related afflictions		In vitro kamahines bioactivity assays *: Herpes simplex virus 1 Poliovirus type 1 Murine leukemia virus	Herpes simplex labialis	Adenovirus Herpes simplex virus 1 Herpes simplex virus 2
Investigated immune-modulatory mechanisms and related afflictions			Regulation on neutrophil respiration burst Inhibition of phagocytose (macrophage) Action on NADPH oxidase Free radical scavenging immuno-modulatory effects on macrophages Pro- or anti-inflammatory properties Afflictions: Eczema Psoriasis Rosacea	Regulation on neutrophil respiration burst Inhibition of phagocytose (macrophage) Action on NADPH oxidase Free radical scavenging immuno-modulatory effects on macrophages Pro- or anti-inflammatory properties

* Primary source unavailable.

## References

[1] Ministry for Primary Industries, Economic Intelligence Unit (2021). Situation and Outlook for Primary Industries (SOPI). https://www.mpi.govt.nz/dmsdocument/45451-Situation-and-Outlook-for-Primary-Industries-SOPI-June-2021.

[2] Carter D.A., Blair S.E., Cokcetin N.N., Bouzo D., Brooks P., Schothauer R., Harry E.J. (2016). Therapeutic Manuka Honey: No Longer So Alternative. Front. Microbiol..

[3] Bil G. (2016). Between Maori and Modern? The case of Manuka honey. Appreciating Local Knowledge.

[4] Apiculture New Zealand (2021). NZ Honey Market Update June 2021. https://apinz.org.nz/wp-content/uploads/2021/06/Apiculture-New-Zealand-Market-Update-June-2021.pdf.

[5] Alvarez-Suarez J., Gasparrini M., Forbes-Hernández T., Mazzoni L., Giampieri F. (2014). The Composition and Biological Activity of Honey: A Focus on Manuka Honey. Foods.

[6] Girma A., Seo W., She R.C. (2019). Antibacterial activity of varying UMF-graded Manuka honeys. PLoS ONE.

[7] FAO (1981). WHO Codex Alimentarius Commission. Codex Standard for Honey.

[8] Ministry for Primary Industries (2013). Options for Defining Monofloral Mānuka Honey. https://www.mpi.govt.nz/dmsdocument/3509-options-for-defining-monofloral-manuka-honey.

[9] Molan P.C. (1998). The limitations of the methods of identifying the floral source of honeys. Bee World.

[10] Hegazi N.M., Elghani G.E.A., Farag M.A. (2021). The super-food Manuka honey, a comprehensive review of its analysis and authenticity approaches. J. Food Sci. Technol..

[11] Saunders L. (2017). The Manuka & Kanuka Plantation Guide.

[12] Dawson M., Heenan P. (2010). Checklist of Metrosideros cultivars. N. Z. Garden J..

[13] De Lange P.J. (2021). *Metrosideros robusta* Fact Sheet (Content Continuously Updated). New Zealand Plant Conservation Network. https://www.nzpcn.org.nz/flora/species/metrosideros-robusta/.

[14] De Lange P.J. (2021). *Weinmannia racemosa* Fact Sheet (Content Continuously Updated). New Zealand Plant Conservation Network. https://www.nzpcn.org.nz/flora/species/weinmannia-racemosa/.

[15] De Lange P.J. (2021). *Knightia excelsa* Fact Sheet (Content Continuously Updated). New Zealand Plant Conservation Network. https://www.nzpcn.org.nz/flora/species/knightia-excelsa/.

[16] Ngā Tipu Whakaoranga (2021). Māori Plant Use Database. Landcare Research Manaaki Whenua. https://Māoriplantuse.landcareresearch.co.nz/.

[17] Zumla A., Lulat A. (1989). Honey—A Remedy Rediscovered. J. R Soc. Med..

[18] Molan P.C. (1999). Why honey is effective as a medicine. 1. Its use in modern medicine. Bee World.

[19] Molan P.C. (2001). Why honey is effective as a medicine: 2. The scientific explanation of its effects. Bee World.

[20] Hermanns R., Mateescu C., Thrasyvoulou A., Tananaki C., Wagener F.A.D.T.G., Cremers N.A.J. (2020). Defining the standards for medical grade honey. J. Apic. Res..

[21] Vanhanen L.P., Emmertz A., Savage G.P. (2011). Mineral analysis of mono-floral New Zealand honey. Food Chem..

[22] Senanayake M.J. (2006). A Chemical Investigation of New Zealand Unifloral Honeys. Ph.D. Thesis.

[23] Langford V., Gray J., Foulkes B., Bray P., McEwan M.J. (2012). Application of Selected Ion Flow Tube-Mass Spectrometry to the Characterization of Monofloral New Zealand Honeys. J. Agric. Food Chem..

[24] Goss C.H.A. (2009). Indicators of Bioactivity and Floral Origin of New Zealand Honeys. Ph.D. Thesis.

[25] Hyink W. (1998). A Chemical Investigation of Some New Zealand Honeys. Ph.D. Thesis.

[26] Broom S.J., Wilkins A.L., Ede R.M., Lu Y. (1992). Isolation and structural characterisation of Kamahine C: An unusual spiroketal found in a native New Zealand honey. Tetrahedron Lett..

[27] Broom S.J., Wilkins A.L., Lu Y., Ede R.M. (1994). Novel nor-Sesquiterpenoids in New Zealand Honeys. The Relative and Absolute Stereochemistry of the Kamahines: An Extension of the Mosher Method to Hemiacetals. J. Org. Chem..

[28] Broom S.J. (1998). Structural Characterisation and Absolute Stereochemistry of Some Degraded Carotenoids from New Zealand Honeys. Ph.D. Thesis.

[29] Ede R.M., Wilkins A.L. (1993). Novel iVor-Sesquiterpenoids in New Zealand Honeys II. Isolation and Structural Characterisation of Meliracemoic Acid. Tetrahedron Lett..

[30] Spiteri M., Rogers K.M., Jamin E., Thomas F., Guyader S., Lees M., Rutledge D.N. (2017). Combination of 1H NMR and chemometrics to discriminate manuka honey from other floral honey types from Oceania. Food Chem..

[31] Sun Y. (1995). A Chemical Investigation of Some New Zealand Native Honeys. Ph.D. Thesis.

[32] Petchell L.E. (2009). Identification of the Floral Source of New Zealand Honeys. Ph.D. Thesis.

[33] Bong J., Loomes K.M., Schlothauer R.C., Stephens J.M. (2016). Fluorescence markers in some New Zealand honeys. Food Chem..

[34] Molan P.C., Smith I.M., Reid G.M. (1988). A Comparison of the Antibacterial Activities of Some New Zealand Honeys. J. Apic. Res..

[35] Allen K.L., Molan P.C., Reid G.M. (1991). A Survey of the Antibacterial Activity of Some New Zealand Honeys. J. Pharm. Pharmacol..

[36] Brady N., Molan P.C., Bang L. (2004). A survey of non-manuka New Zealand honeys for antibacterial and antifungal activities. J. Apic. Res..

[37] Lu J., Carter D.A., Turnbull L., Rosendale D., Hedderley D., Stephens J., Gannabathula S., Steinhorn G., Schothauer R.C., Whitchurch C.B. (2013). The Effect of New Zealand Kanuka, Manuka and Clover Honeys on Bacterial Growth Dynamics and Cellular Morphology Varies According to the Species. PLoS ONE.

[38] Schmidt C., Eichelberger K., Rohm H. (2021). New Zealand mānuka honey—A review on specific properties and possibilities to distinguish mānuka from kānuka honey. LWT.

[39] Tan S.T., Wilkins A.L., Molan P.C., Holland P.T., Reid M. (1989). A Chemical Approach to the Determination of Floral Sources of New Zealand Honeys. J. Apic. Res..

[40] Tan S.T., Holland P.T., Wilkins A.L., Molan P.C. (1988). Extractives from New Zealand honeys. 1. White clover, manuka and kanuka unifloral honeys. J. Agric. Food Chem..

[41] Stephens J.M., Schlothauer R.C., Morris B., Yang D., Fearnley L., Greenwood D.R., Loomes K.M. (2010). Phenolic compounds and methylglyoxal in some New Zealand manuka and kanuka honeys. Food Chem..

[42] Adams C.J., Manley-Harris M., Molan P.C. (2009). The origin of methylglyoxal in New Zealand manuka (*Leptospermum scoparium*) honey. Carbohydr. Res..

[43] Gannabathula S., Skinner M.A., Rosendale D., Greenwood J.M., Mutukumira A.N., Steinhorn G., Stephens J., Krissansen G.W., Schlothauser R.C. (2012). Arabinogalactan proteins contribute to the immunostimulatory properties of New Zealand honeys. Immunopharmacol. Immunotoxicol..

[44] Gannabathula S., Krissansen G.W., Skinner M.A., Steinhorn G., Schlothauer R.C. (2015). Honeybee apisimin and plant arabinogalactans in honey costimulate monocytes. Food Chem..

[45] Bong J., Loomes K.M., Lin B., Stephens J.M. (2018). New approach: Chemical and fluorescence profiling of NZ honeys. Food Chem..

[46] Beitlich N., Koelling-Speer I., Oelschlaegel S., Speer K. (2014). Differentiation of Manuka Honey from Kanuka Honey and from Jelly Bush Honey using HS-SPME-GC/MS and UHPLC-PDA-MS/MS. J. Agric. Food Chem..

[47] Semprini A., Braithwaite I., Corin A., Sheahan D., Tofield C., Helm C., Montgomery B., Fingleton J., Weatherall M., Beasley R. (2015). Randomised controlled trial of topical kanuka honey for the treatment of acne. BMJ Open.

[48] Braithwaite I., Hunt A., Riley J., Fingleton J., Kocks J., Corin A., Helm C., Sheahan D., Tofield C., Montgomery B. (2015). Randomised controlled trial of topical kanuka honey for the treatment of rosacea. BMJ Open.

[49] Leong A.G., Herst P.M., Harper J.L. (2012). Indigenous New Zealand honeys exhibit multiple anti-inflammatory activities. Innate Immun..

[50] Bean A. (2012). Investigating the Anti-Inflammatory Activity of Honey. Ph.D. Thesis.

[51] Kuehne J. (2016). Anti-Inflammatory Properties of New Zealand Honey. Ph.D. Thesis.

[52] Tomblin V., Ferguson L.R., Han D.Y., Murray P., Schlothauer R. (2014). Potential pathway of anti-inflammatory effect by New Zealand honeys. IJGM.

[53] Fingleton J., Helm C., Tofield C., Weatherall M., Beasley R. (2014). A randomised controlled trial of topical Kanuka honey for the treatment of eczema. JRSM Open.

[54] Fingleton J., Sheahan D., Corin A., Weatherall M., Beasley R. (2014). A randomised controlled trial of topical Kanuka honey for the treatment of psoriasis. JRSM Open.

[55] Mane S., Singer J., Corin A., Semprini A. (2018). Successful Treatment of Actinic Keratosis with Kanuka Honey. Case Rep. Dermatol. Med..

[56] Wilkins A.L., Lu Y. (1995). Extractives from New Zealand Honeys. 5. Aliphatic Dicarboxylic Acids in New Zealand Rewarewa (*Knightea excelsa*) Honey. J. Agric. Food Chem..

[57] Willix D.J., Molan P.C., Harfoot C.G. (1992). A comparison of the sensitivity of wound-infecting species of bacteria to the antibacterial activity of manuka honey and other honey. J. Appl. Bacteriol..

[58] Wilkinson J.M., Cavanagh H.M.A. (2005). Antibacterial Activity of 13 Honeys Against *Escherichia coli* and *Pseudomonas aeruginosa*. J. Med. Food.

[59] Bukhari M. (2014). In Vitro-Studies Relating to Honey as an Alternative Approach to Wound Therapy. Ph.D. Thesis.

[60] Daniels B. (2018). Total Synthesis of Lumazine-Containing Natural Products. Ph.D. Thesis.

[61] Littlejohn E.S.V. (2009). The Sensitivity of Adenovirus and Herpes Simplex Virus to Honey. Ph.D. Thesis.

[62] Nyunza G. (2018). Anthropogenic and climatic factors affecting honey production: The case of selected villages in Manyoni District, Tanzania. J. Agric. Biotech. Sustain. Dev..

[63] Mountford-McAuley R., Prior J., Clavijo McCormick A. (2021). Factors affecting propolis production. J. Apic. Res..

